# A Moderating Role of Social Intelligence and Creativity During Primary Career Exploration in Late Adolescence

**DOI:** 10.1192/j.eurpsy.2023.852

**Published:** 2023-07-19

**Authors:** O. B. Chesnokova, S. M. Churbanova, O. V. Markish

**Affiliations:** Department of Developmental Psychology, Faculty of Psychology, Lomonosov Moscow State University, Moscow, Russian Federation

## Abstract

**Introduction:**

Career self-exploration activities can be defined as orienting oneself in one’s own vocational interests, skills, job, and organizational characteristics and as an important career pre-decision stage (Stumpf et al., 1983). Effective career exploratory behaviors are associated with less anxiety regarding uncertainty and difficulties during first career decision-making (Storme, Celik, 2017; Xu et al., 2014). In Study 1, we showed that productive career self-exploration in adolescence is associated with a high level of tolerance for uncertainty (TU) (Chesnokova, Churbanova et al., 2022).

**Objectives:**

The aim of Study 2 was to compare the social intelligence (SI) and creativity (Cr) of students with high and low level career adapt-abilities applied to present and prospective future career decisions.

**Methods:**

Participants were 67 students (15-17 years old). SI was assessed by O’Sullivan & Guilford’s Tests (1966), Cr was measured by CAP consisted of Test of Divergent Thinking and Test of Divergent Feeling (Williams, 1980). Career adapt-abilities were estimated by CAAS (Savickas & Porfeli, 2012; Pryazhnikov, 2016).

**Results:**

Two contrast clusters based on SI and Cr mediated by TU level were analysed. One cluster included students with high pragmatic motivation concerning career path choice. There were students with high TU, high SI and cognitive Cr and low level of Divergent Feeling who focusing on nearest obstacles like entrance exams and distant professional future (professional revenues, social status and on task-oriented content). They are open to new career experiences and flexible in career choice (at r .56, p .001), but they lack social intelligence’s diplomatic techniques and cognitively complicated interpersonal situational awareness. They mostly rely on academic aptitude and self-efficacy. The second cluster of students was made up of those with low TU, average to low SI, and high levels of divergent feelings. They exhibited less pragmatist motivation and active career exploratory behaviors regarding both immediate and long-term professional paths, professional values, the human aspects of potential careers, the social structure of professional society, and the need to use emotional intelligence when interacting with clients and working in teams. They rely on social support from professional representatives and teachers during their career decision making.

**Conclusions:**

In sum, SI and Divergent feelings may be significant mediators in the development of career adaptability and realistic self-perceived confidence in task-oriented content as well as interpersonal specificity of professional social environments. To reduce stress and anxiety of prospective students, the development of self-efficacy and motivation should be given priority over just academic readiness for exams and narrow professional skills.

**Disclosure of Interest:**

None Declared

